# Editorial: Body composition assessment and future disease risk

**DOI:** 10.3389/fmed.2025.1617729

**Published:** 2025-05-08

**Authors:** Ara Jo, Frank A. Orlando, Arch G. Mainous

**Affiliations:** ^1^Department of Health Services Research, Management and Policy, University of Florida, Gainesville, FL, United States; ^2^Department of Community Health and Family Medicine, University of Florida, Gainesville, FL, United States

**Keywords:** body mass index, obesity, body composition, cohort, bioelectrical impedance

Body composition assessment is a fundamental element of chronic disease prevention, diagnosis, and management in clinical practice. Body composition consists of various components, including adipose tissue, muscle mass, bone mass, height, and organ tissues. It is widely used to examine the relationship between these components and health outcomes such as mortality and disease risk. Understanding body composition provides valuable insights into the structure and function of adiposity, the detection of obesity-associated phenotypes, and the mechanisms of chronic disease development (1). Quantifying body composition advances the impact of each component on disease development and management. For instance, body mass index (BMI, kg/m^2^) is the most common method to identify obesity and its associated chronic diseases in practice.

Obesity is a critical attribute of many chronic diseases. Obesity is defined as abnormal or excessive body fat associated with chronic disease risk (2). BMI is a rule of thumb for estimating obesity in practice due to its simple and convenient nature. However, although BMI is associated with chronic disease risks, the limitations of BMI have become increasingly apparent in terms of accurately measuring body fat mass and regional fat distribution (3, 4). Additionally, using BMI alone to measure body fat can lead to significant misidentification of patient health risk classification.

A large body of literature has found that overweight or obese individuals defined by BMI sometimes demonstrate better survival and outcomes than normal-weight counterparts (5–7). These studies explain this relationship between BMI and survival by highlighting the inaccuracy of BMI as a measurement of body fat mass. Specifically, some evidence indicates that elevated BMI, an indirect measure of body fat, is not as useful in predicting downstream mortality as a direct measure of body fat (8). Rather, despite a higher BMI, individuals who have more muscle mass and lower lean mass tend to show better outcomes (9, 10). On the other hand, the “skinny fat” or “metabolically obese normal weight (MONW)” body type demonstrates detrimental outcomes among the normal BMI population. However, these body types are neglected in preventive care screening services due to BMI being the standard. Nevertheless, MONW is strongly associated with metabolic syndrome, type 2 diabetes, and cardiovascular disease (CVD) due to the increased workload of the heart and cytokine malfunction (4, 11, 12). These findings elucidate the need for more effective methods of body composition measurement for better risk assessment of chronic disease morbidity and mortality.

## Body composition measurements and chronic diseases

Body composition is strongly associated with chronic diseases. Particularly, adipose tissue is a critical risk factor for obesity associated with chronic diseases (13). As alternative measurements of body composition to BMI have been proposed, such as waist circumference (WC), waist-to-hip ratio (WHR), and direct measures of body fat percentage (BF%) or lean body mass, the relationship between body fat and chronic disease risk extends beyond the conventional approach using BMI.

Visceral fat tissue is directly associated with chronic disease, and the visceral fat index may be an effective measurement to predict the risk of chronic diseases. Huang et al. used the visceral adiposity index (VAI), an indirect measure of visceral body fat combining information from WC, BMI, triglycerides, and HDL cholesterol, to calculate sex-specific visceral fat function. They categorized VAI into quartiles and found a non-linear relationship between VAI and prediabetes and diabetes for both sexes. Especially, they identified a significant threshold at 2.10 for VAI in the early detection of prediabetes and diabetes. If an individual surpasses the 2.10 threshold, they may be at higher risk for developing those conditions.

Utilizing various body composition measurements can enhance the prediction of chronic diseases and hospitalization outcomes more effectively. A meta-analysis analyzed 22 studies on the relationship between WHR and myocardial infarction (MI) over the past 20 years (Zhang et al.). The results demonstrated that a higher WHR is positively associated with MI compared to a lower WHR. Interestingly, gender-specific analysis confirmed this association more specifically, showing that WHR had a stronger association with MI among females. Yan and Chen proved a strong association of four different body composition assessments, including BMI, body fat percentage, WC, and hip circumference, as risk factors for non-suppurative otitis media (NSOM) while the study did not specify the types of NSOM. Of those, hip circumference had the highest association with NSOM. Canonico et al. used calf circumference (CC) to evaluate the risk of in-hospital complication risk and in-hospital mortality. As a result, lower CC was associated with a higher risk of in-hospital complication development and death during hospitalization or within 90 days of discharge in frail older patients (Canonico et al.).

Nutritional intake patterns in children and adolescents show complex relationships with different obesity phenotypes, contributing to our understanding of early risk factors for pediatric chronic diseases. Dietary habits showed a higher association with abdominal obesity measurements such as WC or waist-to-height ratio (WHtR) rather than BMI in children and adolescents aged 6 to 18 years old (Yun et al.). More specifically, those with unhealthy nutritional intake reported three times higher abdominal obesity prevalence compared to those with healthy nutrition intake.

## Body composition and adverse outcomes

Reports about an “obesity paradox” exist where higher BMI ranges are protective (14). For example, Li et al. showed a significant association between BMI increments and 29-day mortality in patients with sepsis, indicating that higher BMI is significantly associated with lower mortality. Despite such reports, studies also show that lean mass is a mediator between BMI, adiposity, and patient mortality (9, 15). Therefore, directly measuring body fat may have better utility for measuring outcomes in certain cases. In this regard, Fang et al. found that patients undergoing small cell lung cancer immunotherapy with a higher visceral to subcutaneous fat ratio (VSR) reported a worse response to the therapy than those with a lower VSR. Although unadjusted regression models showed significantly worse overall survival and progression-free survival among patients with sarcopenia or lower skeletal muscle mass compared to those with higher muscle mass, the adjusted models did not show significant outcomes (Fang et al.).

More accurate body composition assessment is particularly important when evaluating older adults experiencing sarcopenia with preserved fat mass. Calf circumference (CC), which serves as a valuable muscle mass marker, was the only significant body composition measurement for all in-hospital mortality, complications, and 90-day mortality compared to hand grip strength and existing clinical frailty among older hospitalized patients (Canonico et al.). Since CC is highly associated with mobility and falls (16), it can provide more valuable insights, demonstrating that age-associated changes in body composition predict adverse outcomes more accurately than static measurements.

Lastly, despite the widespread clinical use of BMI as a cardiovascular disease (CVD) risk factor, there may be limitations when using it with the triglyceride-glucose (TyG) index to capture metabolic risk. More specifically, even though BMI and TyG independently proved to have a significant association with CVD, TyG did not play a role as a mediator of CVD when combined with BMI (Gan et al.).

## Body composition assessment evolution

As measurement technology has evolved, more specific body compositions can be measured with a home-based digital scale. However, concerns have been raised about measurement accuracy and usability in practice. Dual-energy X-ray absorptiometry (DXA) scans are the most accurate measurement tool, but they are not easily used in practice and are expensive to use solely for body composition measurement purposes. Bioelectrical impedance analysis (BIA) is an alternative tool that can be used in practice and at home relatively easily. Despite the lower accuracy of BIA compared to DXA scan, its performance is sufficient compared to DXA scan to reliably be used in clinical practice (17–19).

As technology has advanced, new technologies and methodologies have emerged to measure and utilize body composition in predicting health risks. Machine learning is used to measure comprehensive body composition effectively (20, 21). BF% was generated with a smartphone that uses a three-dimensional scanning application, and the results were reliable compared to DXA scans (Tinsley et al.). It can contribute to assessing multi-dimensional aspects of body composition, from appearance to internal body composition, by utilizing advanced measurement with a smartphone in clinical settings. Furthermore, it may be a more effective, cost-effective way to measure whole body composition for patients.

## Clinical implications and conclusion

The growing evidence connecting body composition to chronic disease risk and outcomes has significant implications for clinical practice. Moving beyond the conventional BMI-centric approaches to health assessment allows more precise risk stratification and personalized intervention and care planning. Clinicians can use direct measures of body composition, such as DXA scan or the more clinically utilizable BIA, to identify high-risk patients, such as those with skinny fat or sarcopenic obesity, which might otherwise be missed by using BMI alone.

As body composition and chronic disease research advance, tools and methods directly measuring body composition will become more accurate and reliable. Such tools should be clinically relevant, like BIA, to be valuable in primary care settings where they can enhance early detection of unfavorable body composition and guide preventive intervention before disease develops, ultimately leading to improved patient outcomes and more effective preventive care.
